# Production of Cold-Active Lipase by Free and Immobilized Marine *Bacillus cereus* HSS: Application in Wastewater Treatment

**DOI:** 10.3389/fmicb.2018.02377

**Published:** 2018-10-23

**Authors:** Sahar W. M. Hassan, Hala H. Abd El Latif, Safaa M. Ali

**Affiliations:** ^1^National Institute of Oceanography and Fisheries, Alexandria, Egypt; ^2^City of Scientific Research and Technological Applications, Alexandria, Egypt

**Keywords:** lipase, Plackett–Burman, immobilization, recycling, wastewater treatment

## Abstract

Lipases are enzymes that have the potential to hydrolyze triacylglycerol to free fatty acids and glycerol and have various applications. The aim of the present study was to isolate and screen marine bacteria for lipase production, optimize the production, and treat wastewater. A total of 20 marine bacterial isolates were obtained from the Mediterranean Sea and were screened for lipase production. All isolates were found to have lipolytic ability. The differences between the isolates were studied using RAPD-PCR. The most promising lipase producer (isolate 3) that exhibited the highest lipolytic hydrolysis (20 mm) was identified as *Bacillus cereus* HSS using 16S rDNA analysis and had the accession number MF581790. Optimization of lipase production was carried out using the Plackett–Burman experimental design with cotton seed oil as the inducer under shaking conditions at 10°C. The most significant factors that affected lipase production were FeSO_4_, KCl, and oil concentrations. By using the optimized culture conditions, the lipase activity increased by 1.8-fold compared with basal conditions. Immobilization by adsorption of cells on sponge and recycling raised lipase activity by 2.8-fold compared with free cells. The repeated reuse of the immobilized *B. cereus* HSS maintained reasonable lipase activity. A trial for the economic treatment of oily wastewater was carried out. Removal efficiencies of biological oxygen demand, total suspended solids, and oil and grease were 87.63, 90, and 94.7%, respectively, which is promising for future applications.

## Introduction

Lipases (triacylglycerol acylhydrolase, EC 3.1.1.3) catalyze the hydrolysis and synthesis of long-chain acylglycerols (Salihu and Alam, 2012; Lajis, 2018). They also catalyze the hydrolysis of triglycerides and reverse reactions (interesterification and esterification) (Saxena et al., 2003) and are involved in aminolysis, acidolysis, and alcoholysis. Lipases are produced by archaea, bacteria, fungi, animals, and plants (Cai-hong et al., 2008). Animal and plant lipases are less stable than those of microbial origin. The production of microbial lipases is more suitable, economic, and safer (Vakhlu and Kour, 2006; Treichel et al., 2010). Lipolytic enzymes secreted by *Bacillus* spp. are of considerable biotechnological importance. *B. alcalophilus*, *B. licheniformis*, *B. pumilus*, and *B. subtilis* are the common bacterial lipase producers (Lindsay et al., 2000; Awad et al., 2015).

The production of biodiesel and biopolymers using lipases has been successfully recognized as a novel biotechnological application; furthermore, they can be used in the assembly of new pharmaceuticals, agrochemicals, and flavor compounds in addition to their various industrial applications (Hasan et al., 2006; Chary and Devi, 2018; Sarmah et al., 2018).

Cold-adapted enzymes are considered as biocatalysts for industrial applications. They are used in the synthesis of fine chemicals, food additives, detergents, and in the bioremediation of contaminated water and soil (Margesin and Feller, 2010; Cavicchioli et al., 2011; Patel and Desai, 2018). The manufacture of lipases with cold-active properties is generally extracellular and is greatly affected by nutritional and physicochemical factors, including inducers, nitrogen source, carbon source, temperature, agitation, pH, inorganic sources, and dissolved oxygen (Joshi et al., 2006; Ephraim et al., 2014). It was stated that *Bacillus* spp. could easily use different nutrient sources. However, there were limited searches for the development of a suitable medium supplemented with natural nutrient sources such as vegetable oils for higher lipolytic capability (Ephraim et al., 2014). Optimization of lipase production was performed in many studies (Park et al., 2013; Anasontzis et al., 2014; Bharathi et al., 2018; Sahoo et al., 2018).

For long term enzyme activity, immobilization meets this requirement. Immobilization proposes several advantages such as recycle, ease in applications, stability, improved control of reactions, and simple removal of the immobilized object from the medium (Betigeri and Neau, 2002). Methods of immobilization can be divided into three categories: encapsulation, non-covalent attachment to a support, or covalent and carrier-free cross-linking (Gabrielczyk et al., 2018).

Presence of oily wastes in the aquatic environment prevents the diffusion of oxygen into water, which causes the death of many aquatic organisms. Lipases and microbial strains represent green alternatives for wastewater treatment and can have an effect on reducing the oil and fat content in the wastewater; they may assist in controlling severe pollution problems caused by wastewater. Many previous studies have reported the use of lipolytic enzymes for wastewater treatment (Sarac and Ugur, 2015; Silva-Bedoya et al., 2016; Hu et al., 2018).

Thus, the main goal of the present study is screening for lipase production by diverse bacteria isolated from marine environment and optimization of production setting by statistical means and immobilization techniques. Furthermore, an experiment for wastewater management using the most promising lipase producer will be investigated.

## Materials and Methods

### Isolation and Primary Screening for Lipase Production by Different Bacterial Isolates

Marine bacteria were isolated from different sites along the Mediterranean Sea, including Eastern Harbor, Al Shatby, and Abu-Qir, and were cultured on nutrient agar plates (pH 7) that were prepared with aged filtered seawater. Media used throughout this study were supplied from Oxoid LTD, England. All the chemicals used were of analytical reagent grade.

Lipolytic capability of the isolated bacteria was screened using modified Tween agar base plates that contained the following (gl^−1^): beef extract, 3; peptone, 5; (50% seawater and 50% distilled water). The medium was amended by 1% Tween 80 (v/v). The development of clearance zone (hydrolysis) was considered as a positive result. The size of the halo-forming zones was measured after 24 h of incubation.

### Secondary Quantitative Screening for Lipase Production by the Different Isolates

Secondary screening was performed by the quantification of lipase activity (Kanwar et al., 2006). All isolates were inoculated in mineral based medium that was supplemented with 1% (v/v) cotton seed oil and were incubated at 10°C. Production of lipase was assayed for different time intervals (24, 48, and 72 h). Production medium contained the following (gl^−1^): yeast extract, 0.5; sucrose, 0.5; NaNO_3_, 0.03; K_2_HPO_4_, 0.01; MgSO_4_, 0.05; KCl, 0.05; and FeSO_4_.7H_2_O, 0.001, adjust pH to 7. Subsequently, centrifugation at 10000 ×*g* for 20 min at 4°C was carried out to collect the cell free supernatant for lipase assay (Jensen, 1983).

### Lipase Assay

Estimation of lipase activity was resolute by the titrimetric technique using cotton seed oil as a substrate (Jensen, 1983). In brief, cotton seed oil was emulsified with Arabic gum in 0.05 mM Tris buffer (pH 7). A volume of 100 μl of the enzyme was added to the emulsion and incubated at 10°C for 30 min. The reaction was blocked by the addition of acetone: ethanol (1:1). The liberated fatty acids were predicted by titration with 0.05 M NaOH using phenolphthalein as an indicator. Lipase activity was articulated as the amount of enzyme essential to hydrolyze 1 mole of fatty acids from triglyceride per minute.

### DNA Isolation and Random Amplification of Polymorphic DNA (RAPD-PCR)

Genomic DNA was extracted using the Thermo Fisher kit. Quantity and quality of DNA was determined by Nanodrop spectrophotometric method (Kirby, 1993). Quality of the DNA was checked by calculating the ratio between OD_260_ and OD_280_. RAPD-PCR was used to differentiate between the bacterial isolates to detect the dissimilarity between the isolates. Reaction mixture of RAPD-PCR contained the following: 50 ng genomic DNA, 1 μl of 1X buffer, 0.2 mM dNTPs, 1 μl of 10 picomole primer, 2 units of Taq DNA polemerase, and RNAse and DNAse free water. RAPD is a multiplex marker system that predictably uses single primer to amplify random DNA fragments. Two general oligonucleotide primers were used for the amplification: 16S F: AGAGTTTGATCMTGGCTCAG, 16S1100R: GGG TTG CGC TCG TTG. PCR consists of the following three steps: denaturation at 94°C for 1 min, annealing at 30°C for 1 min, and extension at 72°C for 1 min. After amplification by PCR, the products were checked in 2% agarose gel. Genetic miscellany was determined as the experimental number of differentiation.

### Molecular Identification of the Most Potent Isolate (HSS)

DNA was isolated and purified using the standard procedure (Sambrook et al., 1989). The region of 16S rRNA was amplified using the universal primers (F: 5′AGAGTTTGATCMTGGCTCAG3′and R: 5′TACGGYACCTTGTTACGACTT3′). The reaction was performed using a template DNA. Sequences of the 16S rRNA genes were obtained from the NCBI database. Multiple alignments based on the most closely related sequences and similarity levels were carried out using the BLAST program^1^. A phylogenetic tree was reconstructed using the Bioedit software.

### Effect of Temperature on Lipase Activity

The effect of temperature on lipase production was determined by incubating the culture flasks at different temperature (10, 25, and 30°C). Lipase assay was performed separately for each experiment.

### Effect of Agitation and Different Inducers on Lipase Activity

Two sets of flasks were used to study the effect of aeration; one was incubated under static conditions and the other was kept under shaking condition at 120 rpm. For investigating the effect of different substrates on lipase production, 1% (v/v) of each sterilized oil, including coconut oil, olive oil, sesame oil, corn oil, and palm oil was added separately to the basal medium for 24 h at 10°C. Lipase assay was performed separately for each experiment.

### Optimization of Culture Conditions for Lipase Production Using Plackett–Burman Experimental Design

Screening for the virtual consequence of different factors on lipase production was performed by applying the Plackett–Burman experimental design (Plackett and Burman, 1946; Yu et al., 2009). Low (−) and high (+) levels of each variable were tested as shown in Table 3. In this experiment, 11 independent variables were screened in 12 combinations that were organized according to the Plackett–Burman matrix (Table 3). Row no. 13 in Table 3 illustrates the control (basal). The following equation was used to estimate the main effect of each variable: Exi = (Mi + – Mi-)/N, where Exi is the variable main effect, Mi + and Mi- are the lipase activities (U/ml) in the trials where the independent variables were shown in low and high concentrations, respectively, and N is the number of trials divided by two. Microsoft Excel was applied to calculate the statistical *t*-values for equal unpaired samples and significance of each variable. Verification test was performed to confirm the validity of the optimized medium.

### Effect of Immobilization on Lipase Production by *B. cereus* HSS

#### Immobilization by Entrapment in Calcium Alginate Beads

Entrapment was performed using 3% sodium alginate as previously described (Eikmeier and Rhem, 1987). Sodium alginate (0.75 g) was dissolved in 23 ml of distilled water, followed by autoclaving at 121°C for 10 min. A volume of 2 ml of *B. cereus* HSS suspension was added to the prepared sodium alginate solution. A volume of 10 ml of the bacterial-alginate combination was drawn by using a sterilized syringe and was introduced into sterile 2% CaCl_2_ solution, and the resultant calcium alginate beads entrapping the bacteria were left in CaCl_2_ solution for about 2 h to solidify. Later, the beads were washed several times with sterile distilled water and used as inoculum for 25 ml of the optimized medium.

#### Immobilization by Adsorption

Bacterial immobilization by adsorption was carried out on different solid supports (luffa pulp, clay, ceramic, sponge, and pumice). Bacterial suspensions (2 ml) were added to 25 ml sterile flasks containing the optimized culture medium and 5 g of each porous support. Both sponge and luffa pulp were cut to pieces of the size 0.5 cm and were washed with water before use. The flasks were incubated under slow shaking condition at 120 rpm for 24 h. Lipase activity was estimated each time and was compared with the free cells.

### Recycling of Adsorbed *B. cereus* HSS

A total of 20 cubes of sponge were added to the optimized medium, inoculated with 2 ml of bacterial suspension, and incubated at 10°C for 24 h, followed by removal of the culture medium and addition of a new sterilized medium (25 ml); subsequently, a new cycle was run. This process was regenerated several times. At the end of each cycle, activity of lipase was determined.

### Efficiency of *B. cereus* HSS in Removing Oil and Grease in Addition to Organic Load in Wastewater

Wastewater samples were collected from the drainage effluent of Misr Petroleum Company, Alexandria, Egypt. A volume of 250 ml of the wastewater sample was introduced into each conical flask, sterilized at 121°C for 20 min, and allowed to cool. The sample was inoculated with *B. cereus* HSS containing 10^6^ CFU/ml and was incubated under shaking conditions at 150 rpm. Later, the samples were aseptically drawn every 24 h for 72 h and analyzed for oil and grease, total suspended solids (TSS), and biological oxygen demand (BOD) before and after bacterial inoculation. Samples without the bacterial inoculum were kept as control (Bala et al., 2014). The estimation of each parameter was done according to the method described by Clesceri et al. (1999).

The efficiency for organic load reduction was calculated as follows:

Efficiency % (BOD) removal = (Initial (BOD) of the raw sample -final (BOD) after treatment/ Initial (BOD) of the raw sample) ^∗^100
Efficiency % (TSS) removal = (Initial TSS) of the raw sample -final (TSS) after treatment/ Initial (TSS) of the raw sample) ^∗^100
Efficiency % (oil) removal = (Weight of crude oil before treatment (initial) – Weight of crude oil after treatment)/ Weight of crude oil initial)^∗^100

### Analysis of Residual Oil by Gas Chromatography-Mass Spectrometry (GC-MS)

The residual oil was extracted by dichloromethane. The aqueous phase sample was discarded, and the crude oil containing the solvent was concentrated to approximately 0.1 ml using a rotary evaporator under reduced pressure. The residual oil was subjected to chemical analysis using gas chromatography-mass spectrometry (GC-MS; Agilent Technologies 7890A GC System with a flame ionization detector, a 5975C inert XL MSDTriple-Axis Mass Detector and Agilent 19,091S-433 Trace Analysis column). The GC experiments were conducted by injecting 1 μL of the sample, operating in the split less mode with an evaporation temperature of 250°C, pressure of 1.8 bar, and flow rate of 2.5 ml/min. Helium carrier gas temperature was maintained in gradients of 50°C/min, 40°C/min, 300°C/min, and 300°C/5 min. Compounds were characterized based on the similarities between their mass spectrum and those of the authentic data presented by Wiley Library.

## Results and Discussion

### Qualitative Screening for Extracellular Lipase Production by the Isolated Bacteria

**FIGURE 1:**
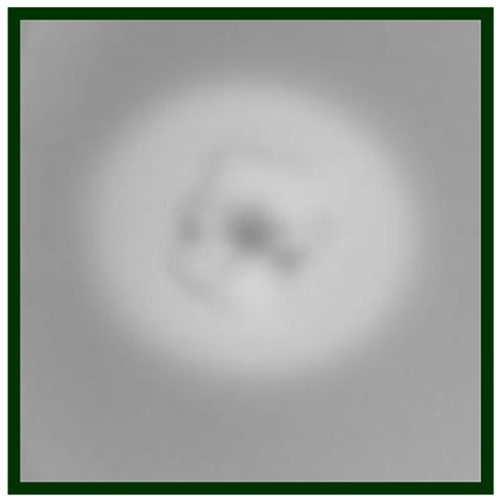
Hydrolytic zone of Tween 80 produced by isolate 3.

The capability of all isolates to degrade Tween 80 was tested using Tween agar base medium. Lipase-producing potentiality was investigated based on the size of the clear zone around each colony (Ramle and Abdul Rahim, 2016). Results indicated that all isolates were positive producers of lipase with different efficiency as the lipolytic zone diameter range was 6–20 mm. The highest lipolytic activity was detected for isolate 3, which recorded 20 mm (Figure 1) followed by strain 10 (17 mm), whereas strains 15, 16, and 19 showed the lowest lipolytic activity (6 mm) as shown in Table 1. Tween has been used as a substrate in different studies and is recommended as it is more easy and convenient for the detection of lipase activity (Anbu et al., 2011). Lo Giudice et al. (2006) studied the effectiveness of different isolated bacteria for their lipolytic activity on Tween and tributyrin by hydrolytic plate assay, and they reported that among the strains tested for lipid hydrolysis, only 7 isolates were unable to utilize the media, while the remaining hydrolyzed at least one substrate, and the diameters of the halos were generally 9–29 mm using Tween. In a parallel study, Rasmey et al. (2017) reported that among the 56 tested isolates, only 24 isolates were recorded as lipase producers with lipolytic zone diameters of 18–32 mm.

**Table 1:** Screening of lipolytic activities of the isolated bacteria.

Isolates	Colony diameter (mm)	Lipolytic zone diameter (mm)
1	6	12
2	5	7
3	3	20
4	4	9
5	4	8
6	5	12
7	3	13
8	3	8
9	3	15
10	4	17
11	4	14
12	5	10
13	5	10
14	4	12
15	3	6
16	4	6
17	4	15
18	7	11
19	4	6
20	3	14

### Quantitative Screening of Lipase Production by the Isolated Bacteria

Secondary screening was carried out on the isolated bacteria to ensure the capability for lipase production in mineral based medium supplemented with 1% (v/v) cotton seed oil. Lipase production by the selected isolates was estimated at different time intervals (24, 48, and 72 h). As shown in Table 2, the highest production of lipase (225 U/ml) was recorded for strain 3 after 24 h; thus, it was selected for further study. In a parallel study by Rasmey et al. (2017), it was reported that the highest lipase activity (20.0 ± 0.29 U/mL) was recorded for *Pseudomonas monteilii* using olive oil as the substrate in the medium. Similarly, the highest lipase activity (2.33 ± 0.12 U) was indicated by *Staphylococcus* sp. in the presence of cotton seed oil as the carbon source (Patel et al., 2018).

**Table 2:** Time course production of lipase by the isolated bacteria.

Strain number	Lipase activity (U/ml) in different time intervals (h)		
	24	48	72
1	210	200	50
2	75	75	100
3	225	105	125
4	150	150	75
5	125	150	110
6	200	175	135
7	75	215	220
8	125	100	75
9	175	125	65
10	175	200	200
11	100	100	75
12	100	100	75
13	150	200	210
14	200	210	250
15	0	75	150
16	0	100	50
17	100	185	75
18	125	125	175
19	50	125	150
20	170	220	125

### Detection of Polymorphism Among Lipase Producers Using RAPD-PCR

**FIGURE 2:**
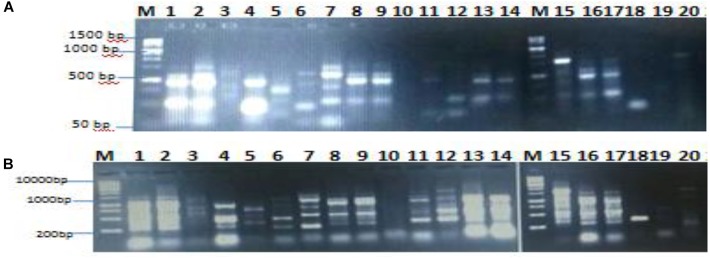
Agarose gel shows polymorphism between isolates using primer 16S F **(A)** and 16S1100R **(B)**.

The genetic analysis of the isolated lipase producers was carried out with the main goal of identifying duplications of the same isolates. RAPD-PCR is the most commonly used marker to detect polymorphism and variability (Shahid et al., 2012; Sobhakumari, 2012). It is easy, quick, and requires small quantity of template DNA. In the present study, isolates coded 1–20 were exposed to DNA extraction and RAPD-PCR with the aim to differentiate between the bacterial isolates. As shown in Figures 2A,B, there was clear polymorphism between the isolates, which was identified using different primers namely 16S1100R and 16S F. RAPD-PCR profiling dependent polymorphism was previously used to differentiate between lipase producing bacterial isolates (Tilwari et al., 2016).

### Molecular Identification of the Most Promising Isolate

**FIGURE 3:**
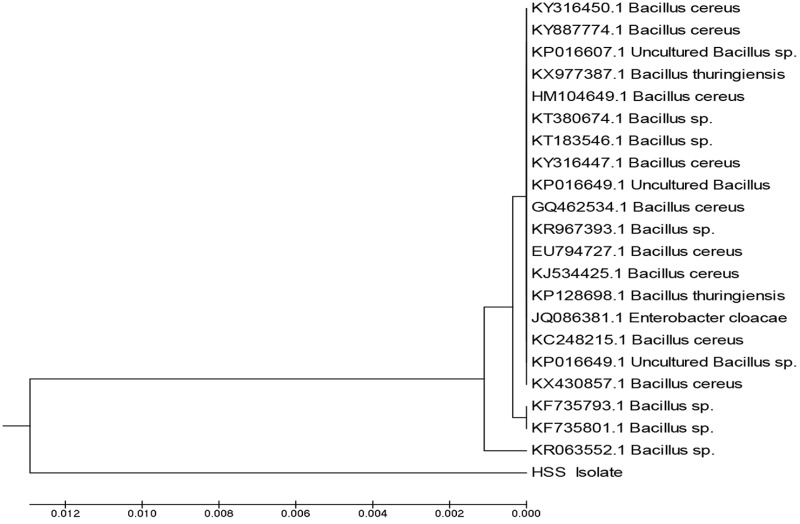
Phylogenetic tree showing the incidental evolutionary relationships between different biological *Bacillus* species. The dendrogram was generated using the MEGA 5 program.

The comparison of 16S rRNA gene sequence of an isolate with the sequences of the different types of strains provides a powerful tool to classify and identify prokaryotes (Kim and Chun, 2014; Zuppa et al., 2014). In the current study, DNA of isolate 3 was extracted and 16S rRNA gene was amplified; the amplicons produced were analyzed using agarose gel electrophoresis. The sequence was deposited in GenBank and had the accession number MF581790; the isolate was identified as *Bacillus cereus* HSS with a similarity of 98%. Figure 3 represents the phylogenetic tree showing the incidental evolutionary relationships among different *Bacillus* species. *Bacillus* species were previously reported as lipase producers in various studies (Bharathi et al., 2018; Femi-Ola et al., 2018; Sahoo et al., 2018).

### Optimization of Fermentation Factors Affect Lipase Production

#### Effect of Temperature on Lipase Production

**FIGURE 4:**
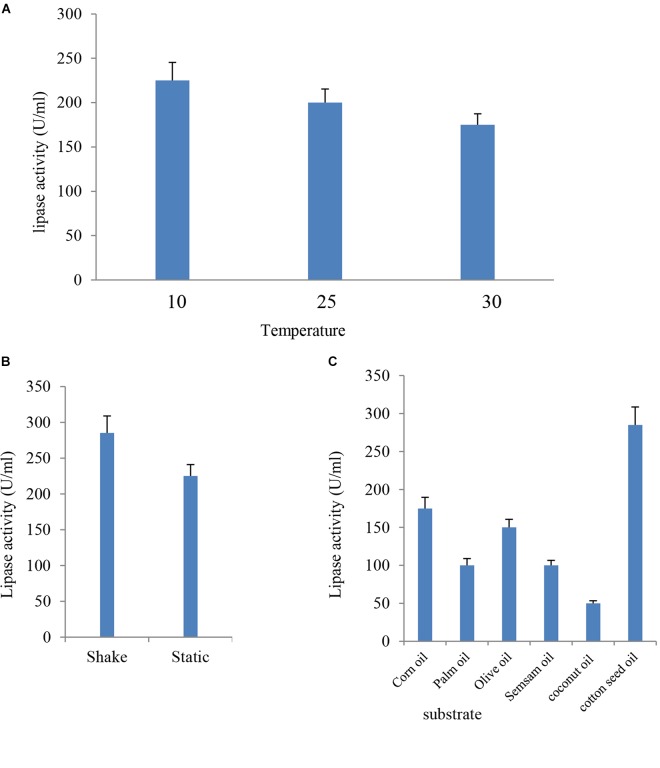
Effect of temperature **(A)**, agitation **(B)**, and different inducer substrates **(C)** on lipase activity of *B*. *cereus* HSS.

The culture media were incubated at different temperatures (10, 25, and 30°C) under static conditions for the optimum incubation time. Results indicated in Figure 4A showed that the most suitable temperature for the highest lipase activity (225 U/ml) was 10°C, whereas the lowest lipase activity (175 U/ml) was detected at 30°C. These findings were consistent with previous studies, where lower fermentation temperature had a positive effect on lipase production (Anasontzis et al., 2014) Among the tested range of temperature (15–35°C), the optimum temperature for cold-active lipase production by *Pichia lynferdii* NRRL Y-7723 was 20°C as previously reported (Park et al., 2013). The effect of temperature on enzyme activity is attributed to its effect on stability, rate of the reaction, substrate solubility as well as direct influence on esterification reaction (Musa et al., 2018).

#### Effect of Agitation on Lipase Production by *B. cereus* HSS

Lipase production was premeditated under the effect of agitation at 10°C for 24 h. Data presented in Figure 4B showed that lipase activity reached a value of 285 U/ml, which represented a 1.3-fold increase at shaking condition when compared with the (225 U/ml) static condition. Aeration has up-and-down effects on lipase production by different organisms (Gilbert et al., 1991). Enzyme activity had a significant elevation under agitation when compared with the stationary state. Enzyme production increased when the agitation rate increased up to 150 rpm, and, subsequently, a reduction in activity was reported (Sooch and Kauldhar, 2013). The decrease in enzyme activity at elevated agitation rates might be attributed to the depletion in the nutrient level and oxygen in the medium and the shared stress on bacterial cells in addition to enzyme structure (Calderbank and Moo-Young, 1959).

#### Effect of Different Inducers on Lipase Production by *B. cereus* HSS

Lipases are inducible enzymes. Glycerol, hydrolysable esters, fatty acids, bile salts, Tweens, and triacylglycerols take action as inducers for lipase production (Griebeler et al., 2011). In the present study, sesame oil, corn oil, olive oil, palm oil, coconut oil, and cotton seed oil were tested as inducers for lipase production by *B. cereus* HSS; the enzyme appeared most active toward cotton seed oil with an enzyme activity of 285 U/ml. In contrast, the lipase attacked olive oil, corn oil, palm oil, sesame oil, and coconut oil more slowly (Figure 4C). Cotton seed oil is a cheap, commercial, and available economic source for lipase production. Therefore, it was previously used as a carbon source for lipase production (Abd-Elnaby et al., 2015). The current study indicated that the use of different lipids caused variations in lipase activity. These results are in harmony with the findings of El-Sawah et al. (1995). It was reported in many published experimental data that natural oils stimulate lipase production (He and Tan, 2006; Kaushik et al., 2006). Similarly, elevated level of lipase production by *Bacillus sp*. was observed upon using olive oil as substrate in the custom medium (Eltaweel et al., 2005).

El-Sawah et al. (1995) tested the effect of different substrates that included olein, sunflower, coconut, olive oil, soybean, cotton seed, stearin, butter, castor, and tallow fat, which were appended to the basal medium at a concentration of 1%. It was shown that olive oil was the most favorable substrate for the increased production of lipase by *Lactobacillus delbrueckii* subsp. *bulgaricus*.

Divya et al. (2015) studied lipase production by cold-adapted *Geotrichum* sp. and *Rhodotorula* sp. with a wide range of commercial oils like sunflower oil, olive oil, coconut oil, vanaspati, and ghee at 30°C and reported that palm oil was the most preferable substrate for maximum lipase production.

### Optimization of Culture Conditions Using Plackett–Burman Experimental Design

**FIGURE 5:**
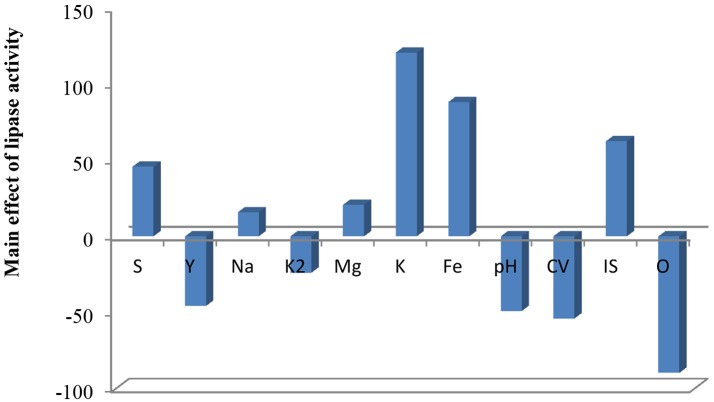
Elucidation of fermentation conditions affecting the production of lipase.

Plackett–Burman experimental design was used with 11 different fermentation conditions. The examined factors and their levels are presented in Table 3. The average results of duplicate experiments are given as the response (U/ml) (Table 3). The estimated main effect of each variable on lipase production and *t*-values were shown in Table 3 and Figure 5.

**Table 3:** Levels of examined factors and statistical analysis of the Plackett–Burman experimental design.

Trial	Factor symbol											Lipase activity (U/ml)

	S (g/l)	Y	Na	K2	Mg	K	Fe	pH	CV	IS	O	
		
			(g/l)					pH	(ml)	(%)	(%)	
**1**	+ [0.8]	[1. 5]	+ [0.08]	+[0.03]	+ [0.08]	+[0.08]	+ [0.002]	+[8]	+ [75]	+[2]	+ [2]	225
**2**	−[0.2]	+ [1.5]	−[0.03]	+ [0.03]	+[0.08]	+ [0.08]	−[0.001]	−[6]	−[25]	+ [2]	−[0.5]	200
**3**	−[0.2]	−[0.5]	+ [0.08]	−[0.01]	+ [0.08]	+[0.08]	+ [0.002]	−[6]	−[25]	−[0.5]	+ [1]	535
**4**	+ [0.8]	−[0.5]	−[0.03]	+ [0.03]	−[0.05]	+ [0.08]	+[0.002]	+ [8]	−[25]	−[0.5]	−[0.5]	275
**5**	−[0.2]	+ [1.5]	−[0.03]	−[0.01]	+ [0.08]	−[0.05]	+ [0.002]	+[8]	+ [75]	−[0.5]	−[0.5]	150
**6**	−[0.2]	−[0.5]	+ [0.08]	−[0.01]	−0.05]	+ [0.08]	−[0.001]	+ [8]	+[75]	+ [2]	−[0.5]	125
**7**	−[0.2]	−[0.5]	−[0.03]	+ [0.03]	−[0.05]	−[0.05]	+ [0.002]	−[6]	+ [75]	+[2]	+ [2]	250
**8**	+ [0.8]	−[0.5]	−[0.03]	−[0.01]	+ [0.08]	−[0.05]	−[0.001]	+ [8]	−[25]	+ [2]	+[2]	75
**9**	+ [0.8]	+[1.5]	−[0.03]	−[0.01]	−[0.05]	+ [0.08]	−[0.001]	−[6]	+ [75]	−[0.5]	+ [2]	225
**10**	+ [0.8]	+[1.5]	+ [0.08]	−[0.01]	−[0.05]	−[0.05]	+ [0.002]	−[6]	−[25]	+ [2]	−[0.5]	185
**11**	−[0.2]	+ [1.5]	+[0.08]	+ [0.03]	−[0.05]	−[0.05]	−[0.001]	+ [8]	−[25]	−[0.5]	+ [2]	100
**12**	+ [0.8]	−[0.5]	+ [.0.08]	+[0.03]	+ [0.08]	−[0.05]	−[0.001]	−[6]	+ [75]	−[0.5]	−[0.5]	100
**13**	0[0.5]	0[1]	0[0]	0[0]	0[0.03]	0[0]	0[0]	0[7]	0[50]	0[1]	0[1]	285
**Main effect**	45.8	−45.8	15.8	−24	20.8	120.8	88.3	−49.2	−54.2	62.5	−90	
***t*-value**	0.629	−0.629	0.213	−0.327	0.281	1.895	2.16	−0.677	−0.749	−0.749	−1.32	


*S: Sucrose; Y: Yeast extract; Na: NaCl; K_2_: K_2_HPO_4_ ; Mg: MgSO_4_; K: KCl; Fe: FeSO_4_; CV: Culture volume; IS: Inoculum size; O: Oil concentration.*

**FIGURE 6:**
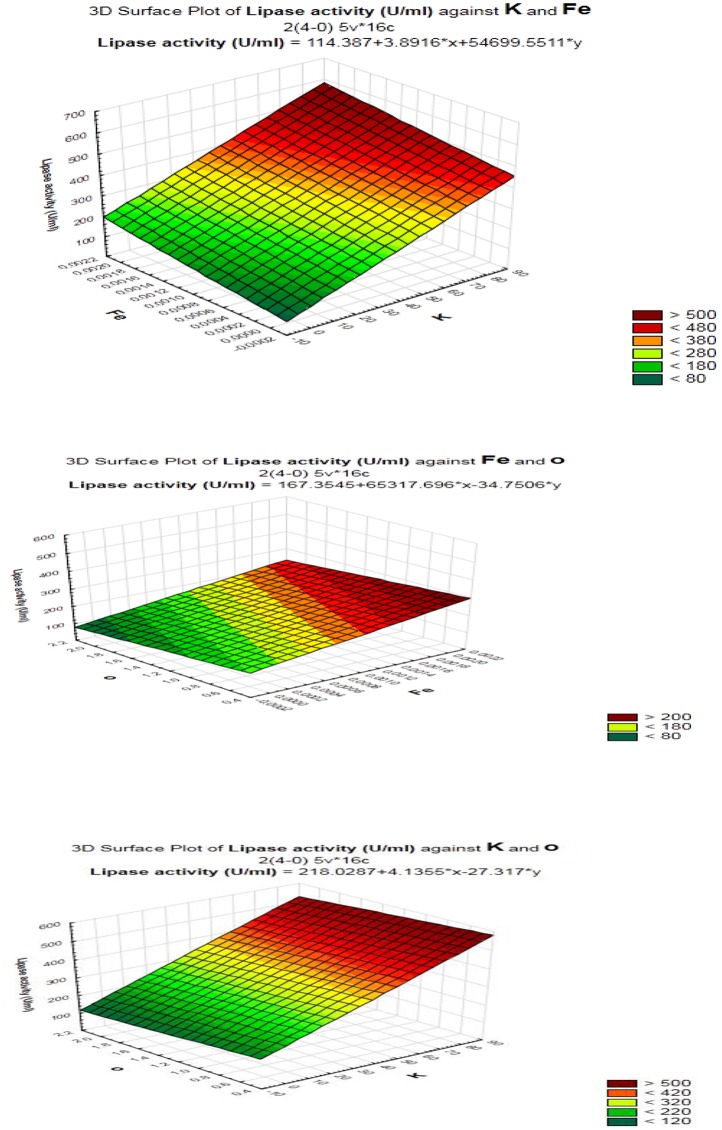
Response surface plots of the interaction between process variables in lipase production by *B. cereus* HSS.

As shown (Figure 5), positive effects of sucrose, NaCl, MgSO_4_, KCL, FeSO_4_, and inoculum size on lipase production were observed, which means that high levels of these parameters will correspond to higher lipase activity, whereas yeast extract, K_2_HPO_4_, pH, culture volume, oil concentration had negative effects. Thus, the predicted optimum medium that resulted from this experiment included the following (g/l) sucrose, 0.8; yeast extract, 0.2; K_2_HPO_4_, 0.01; MgSO_4_, 0.08; and KCl, 0.08; pH, 6; culture volume, 25; oil concentration, 0.5%. Data analysis using *t*-test (Table 3) revealed that FeSO_4_, KCl, and oil concentrations were the most significant factors that affected lipase activity. Interactions among the three factors are illustrated (Figure 6). Many authors applied Plackett–Burman experimental design for the enhancement of enzyme production (Collaa et al., 2016; Kai and Peisheng, 2016; Vasiee et al., 2016; Ebrahimipour et al., 2017; Mazhar et al., 2017).

**FIGURE 7:**
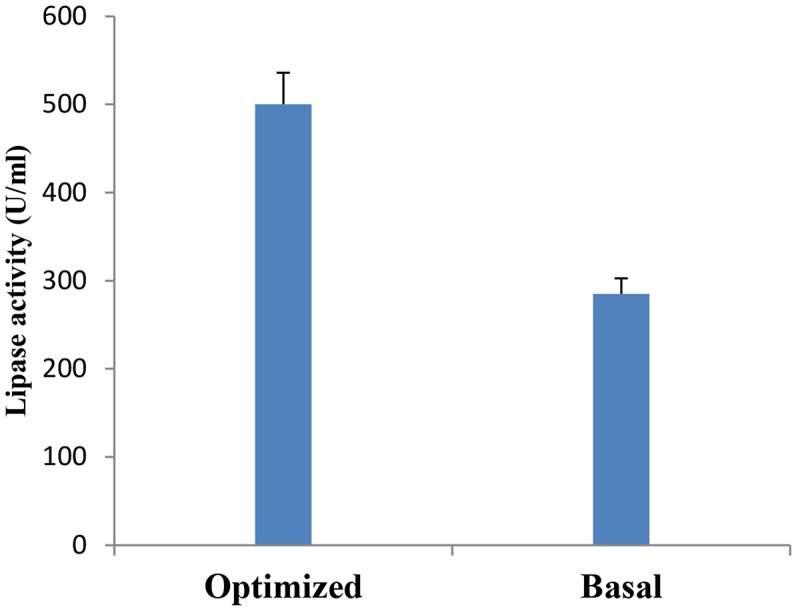
Verification experiment: Lipase production by *B. cereus* HSS grown on basal versus optimized medium.

A verification test was carried out to validate the results gained from the statistical analysis of Plackett–Burman design, using the predicted optimized media against the basal condition media. As shown in Figure 7, lipase activity reached a value of 500 U/ml with an 1.8-fold increase when grown in the optimized medium compared with the basal condition. In comparable studies conducted by Kai and Peisheng (2016) and Sharma et al. (2009), 1.85 and 1.6 fold increases, respectively, were achieved in lipase production upon the application of the Plackett–Burman experimental design for medium optimization.

Another study by Maharana and Ray (2014) showed that the formulated medium predicted by the Plackett–Burman design enhanced lipase production by Pseudomonas sp. Akm-L5 at 15°C by 3.2-fold.

### Effect of Immobilization on Lipase Production by *B. cereus* HSS

**FIGURE 8:**
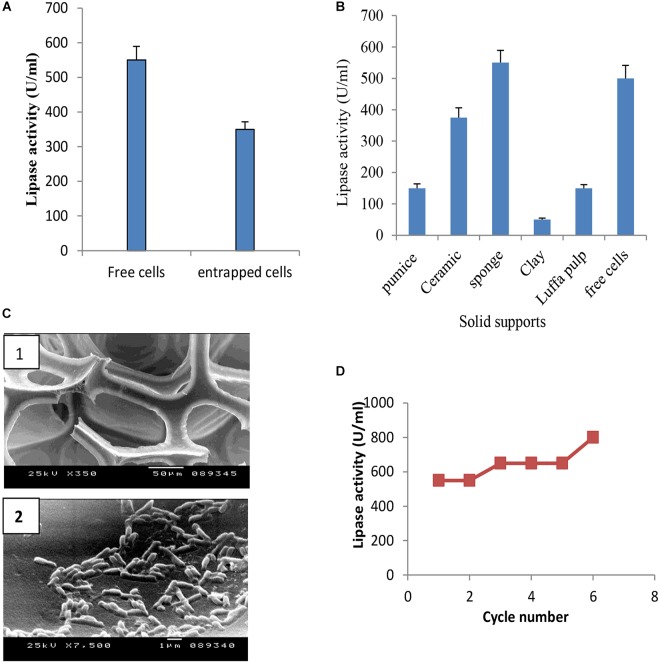
Production of lipase by entrapped cells **(A)** and adsorbed cells on different support materials **(B)**, electron micrograph of sponge **(C1)** and cells adsorbed on sponge **(C2)**, and recycling of adsorbed *B. cereus* HSS on sponge **(D)**.

Immobilization of microbial cells could eliminate the time consuming, tedious, and costly steps involved in the isolation and purification of intracellular enzymes. It also intends to improve the stability of the enzyme (Chandorkar et al., 2014; Lee et al., 2017). To study the effect of entrapment on lipase activity, entrapment of *B. cereus* HSS cells was carried out in 3% sodium alginate. Results (Figure 8A) showed that entrapped cells exhibited lower lipase activity when compared with free cells. Adsorption of cells was carried out using luffa pulp, pumice, clay, ceramic, and sponge as support materials. The adsorbed cells were tested for their potentiality in the production of lipase. As shown in Figure 8B, lipase activity was decreased by 1.5, 3.3, 11 and 3.7 fold upon using ceramic, pumice, clay, and luffa, respectively. The productivity obtained by the immobilized cells on sponge was considerably higher than the free cells. A comparative study indicated that the dramatic variation and the efficiency of an immobilization process rely on the support used (Musa et al., 2018). Thus, cells adsorbed on sponge (Figure 8C) were chosen to complete the study, when these immobilized cells were reused for five successive cycles. As shown in Figure 8D, lipase activity increased in the first cycle and was found to be nearly constant from the second to the fourth cycles, followed by an increase in the fifth cycle to reach a value of 800 U/ml. Accordingly, recycling of *B. cereus* HSS cells on sponge revealed increases of 1.5-fold and 2.8-fold when compared with free cells grown in the optimized and basal conditions, respectively.

It was reported that in comparison with free suspended cells, immobilized cells exhibit tolerance to toxic substrates, enhance fermentation productivity, can adapt to a wide range of pH environments and high process temperature, and are reusable (Jaiswal and Joseph, 2011).

### Efficiency of *B. cereus* HSS in Wastewater Treatment

The present experiment was a trial for the reduction of contaminants in wastewater by the marine lipase producer *B. cereus* HSS. To achieve this goal, BOD, TSS, and oil and grease were estimated before and after the treatment of wastewater with *B. cereus* HSS. Results mentioned in Table 4 showed that the highest reduction efficiencies of BOD, TSS, and oil and grease were 87.63, 90, and 94.7%, respectively, after 72 h. These results confirmed the reduction efficiency of the tested parameters at low temperature, which is considered to be feasible and economic, representing a low energy biological method compared with the traditional methods that require high temperature and energy levels. In a similar study, El Bestawy et al. (2005) confirmed the efficiency of biological treatment of wastewater using the lipase producer *Pseudomonas* spp. individually or in combination. The same finding was also reported in another study (Bala et al., 2014).

**Table 4:** Removal efficiency percentages of BOD, TSS, and oil and grease after treatment by the lipase producer marine *B. cereus* HSS.

Incubation time (h)	Removal efficiency (%) of BOD	Removal efficiency (%) of TSS	Degradation efficiency (%) of oil& grease
24	10	60	15
48	22	87.2	40
72	87.63	90	94.7

### GC-MS Analysis of the Residual Oil

**FIGURE 9:**
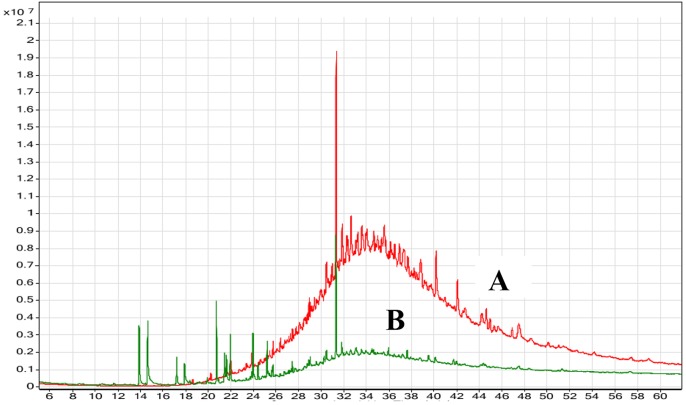
GC-MS chromatogram of the oily wastewater **(A)** control (untreated) and **(B)** treated with lipase producer *B. cereus* HSS after 72 h.

The degradation of oily wastes by marine *B. cereus* HSS after 72 h of incubation was confirmed using GC-MS analysis and was compared with the control (untreated waste). Chromatographic profile of the extracted oil (Figure 9) showed a remarkable decrease in the concentration of fatty acids (*n-*_C12_-*n-*C_23_). On the other hand, complete removal of other compounds (n-C_25_-n-C_32_) was detected after 72 h. Shon et al. (2002) confirmed the removal efficiency of fat, oil, and grease by *Pseudomonas* sp. strain D2D3. Another study found that the biological addition of microorganisms to wastes showed efficiency in removing fat, oil, and grease from wastewater (Kang, 1988).

## Conclusion

The current research explored our local environments, searching for marine bacterial isolates that produce lipase. It suggested that *B. cereus* HSS isolated from the local marine habitat had the potentiality for lipase production. Optimization of the fermentation conditions and the medium components was carried out using one factor at a time experiment, Plackett–Burman experimental design and immobilization, indicating a 2.8-fold increase in lipase activity. The marine isolate *B. cereus* HSS would be potentially useful for the removal of grease traps to treat oily wastewaters, which would represent an economic alternative for wastewater treatment. Large scale applications in waste treatment need to be conducted in the future work.

## Author Contributions

SH conceived, designed, and coordinated the study. SH, HAEL, and SA carried out the experiments. All authors read and approved the final manuscript.

## Conflict of Interest Statement

The authors declare that the research was conducted in the absence of any commercial or financial relationships that could be construed as a potential conflict of interest.

## Footnotes

1 http://www.ncbi.nlm.nih.gov/blast
